# A Dual GLP-1/GIP Receptor Agonist Is More Effective than Liraglutide in the A53T Mouse Model of Parkinson's Disease

**DOI:** 10.1155/2023/7427136

**Published:** 2023-09-25

**Authors:** Zijuan Zhang, Ming Shi, Zhengmin Li, Yuan Ling, Luke Zhai, Ye Yuan, He Ma, Li Hao, Zhonghua Li, Zhenqiang Zhang, Christian Hölscher

**Affiliations:** ^1^School of Medical Sciences, Henan University of Chinese Medicine, Zhengzhou 450046, Henan, China; ^2^Academy of Chinese Medical Sciences, Henan University of Chinese Medicine, Zhengzhou 450046, Henan, China

## Abstract

Parkinson's disease (PD) is a complex syndrome with many elements, such as chronic inflammation, oxidative stress, mitochondrial dysfunction, loss of dopaminergic neurons, build-up of alpha-synuclein (*α*-syn) in cells, and energy depletion in neurons, that drive the disease. We and others have shown that treatment with mimetics of the growth factor glucagon-like peptide 1 (GLP-1) can normalize energy utilization, neuronal survival, and dopamine levels and reduce inflammation. Liraglutide is a GLP-1 analogue that recently showed protective effects in phase 2 clinical trials in PD patients and in Alzheimer disease patients. We have developed a novel dual GLP-1/GIP receptor agonist that can cross the blood-brain barrier and showed good protective effects in animal models of PD. Here, we test liraglutide against the dual GLP-1/GIP agonist DA5-CH (KP405) in the A53T tg mouse model of PD which expresses a human-mutated gene of *α*-synuclein. Drug treatment reduced impairments in three different motor tests, reduced levels of *α*-syn in the substantia nigra, reduced the inflammation response and proinflammatory cytokine levels in the substantia nigra and striatum, and normalized biomarker levels of autophagy and mitochondrial activities in A53T mice. DA5-CH was superior in almost all parameters measured and therefore may be a better drug treatment for PD than liraglutide.

## 1. Introduction

Parkinson's disease (PD) is a chronic neurodegenerative disorder that is clinically identified by typical motor symptoms (rigor, tremor, and akinesis) and characterized by progressive dopaminergic neuronal loss in the substantia nigra pars compacta (SNpc), which leads to loss of striatal dopaminergic synaptic transmission [1, 2]. A key pathological feature is the aggregation of the peptide alpha-synuclein (*α*-syn) and the development of chronic inflammation in the CNS that leads to the release of proinflammatory cytokines and neurodegeneration [3–5]. This can lead to impairment of growth factor signalling, mitochondrial dysfunction, and insulin desensitization [6–9]. Glucagon-like peptide 1 (GLP-1) is a peptide hormone that can resensitize insulin signalling, and GLP-1 mimetics are widely used in treating type 2 diabetes [10–12]. GLP-1 receptor agonists have shown good effects in animal models of PD [13]. Importantly, the GLP-1 receptor agonist exendin-4 (Exenatide, Byetta, Bydureon) has shown impressive protective effects in a phase II trial in PD patients by halting disease progression [14, 15] and resensitized insulin signalling in the brain [16]. Liraglutide is a long-lasting GLP-1 analogue and is available in the market to treat type 2 diabetes [17]. It previously showed good neuroprotective effects in animal models of PD [18–20]. In a phase II clinical trial in PD patients, liraglutide showed clear improvements. Disease progression was much reduced and motor controlled, and the quality of life assessment was improved [21]. Glucose-dependent insulinotropic polypeptide (GIP) is a peptide hormone from the same family as GLP-1 [22]. GIP receptor agonists have shown good protective effects in animal models of PD [23, 24]. GIP acts in synergy with GLP-1, and dual GLP-1/GIP receptors have been developed that show good effects in animal models of PD [13, 25, 26]. We have developed dual agonists that have been modified to cross the blood-brain barrier (BBB) at an enhanced rate. These dual agonists have shown superior neuroprotective effects in animal models of PD [13, 26–30]. BBB penetration is essential for a drug to be effective in treating PD. A recent phase II trial that tested a PEGylated version of exendin-4 in PD did not show any effects [31]. The drug called NLY01 does not cross the BBB readily and therefore shows only limited effects in the clinic [32]. *α*-Synuclein is a peptide that is a biomarker for PD and is seen as one of the drivers of the disease [33–35]. Mutations of the human *α*-synuclein gene can lead to early onset PD [36, 37]. A standard animal model for PD is the A53T-mutated human *α*-synuclein gene expression model [38, 39]. This mouse model expresses the human A53T-mutated *α*-synuclein gene and is a standard animal model of synucleinopathy in the brain. We, therefore, tested the novel dual GLP-1/GIP receptor agonist DA5-CH that previously showed good effects in the MPTP mouse model and the 6-OHDA rat model of PD [29, 32, 40] in the transgenic A53T mouse model of PD and compared the effects with those of liraglutide.

## 2. Materials and Methods

### 2.1. Peptides and Chemicals

The peptides DA5 and liraglutide were synthetized by ChinaPeptides Co., Ltd. (Shanghai, China) with 95% purity. The sequence of DA5 is YXEGTFTSDYSIYLDKQAAXEFVNWLLAGGPSSGAPPPSKRRQRRKKRGY-NH2 (*X* = aminoisobutyric acid). The purity of the peptide was analyzed by reversed-phase high HPLC and characterized using matrix-assisted laser desorption/ionisation time of flight (MALDI-TOF) mass spectrometry.

### 2.2. Animals

We used A53T transgenic mice (Jackson Laboratories, USA). Animals were kept in SPF conditions, and the cage temperature was maintained at 20–24°C, with relative humidity 45%–60% and 12 h light/12 h dark cycle. All the animals had feed and water ad-lib. Mouse DNA was extracted and genotyped using the K9053 kit (Nantome Biotech Co. Ltd.,), see Jackson Lab website protocol for details (https://www.jax.org/strain/004479). Homozygous mice were selected and mated [38]. The Animal Care and Use Committee of Henan University of Chinese Medicine (no: DWLL201903076) approved all procedures.

**Figure 1:**
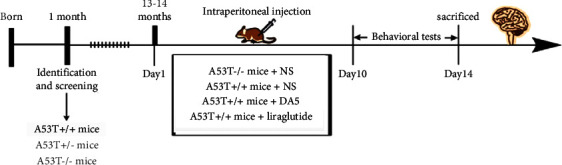
Study design of the test of DA5-CH with liraglutide as a comparator. Both drugs were tested at 25 nmol/kg and 50 nmol/kg bw ip. once daily. Control mice received normal saline (NS).

Both male and female A53T transgenic homozygous mice were used in this study and divided into three groups of 12 mice each. Animals were at 13-14 months of age, and age-, sex-, and A53T genotype-matched negative littermates were used as a control group. The *N* control group included A53T (−) mice and intraperitoneal injection of saline; the A53T group included A53T (+) mice, and saline administration; the CCK group included A53T (+) mice and intraperitoneal injection of DA5-CH; the Lira group included A53T (+) mice and intraperitoneal injection of liraglutide. DA5 was injected daily intraperitoneally at 25 nmol/kg or with liraglutide injection at 50 nmol/kg. All drugs and saline were administered for 14 consecutive days, injecting once daily, see Figure 1 for details.

### 2.3. Behavioral Tests

#### 2.3.1. Rotarod Test

The mice were familiarized with the apparatus (RWD Life Technology, Shenzhen, China) daily for 3 days before the test and trained for 3 minutes daily with a speed at 20 rpm. In the testing condition, the speed was set to 20 for 20 seconds and then accelerated to 30 for 30 seconds. The latency to fall off the rotating rod was recorded. Mice were tested 6 times.

#### 2.3.2. Open Field Test

To assess the motor activity and spontaneous exploratory activity of PD mice, open field tests were conducted. The open field consisted of a square arena (45 × 45 cm) with 25 cm high opaque walls. The floor was divided into 9 equal squares. The central square was defined as the central square (15 × 15 cm). Mice were placed in the center of the apparatus. The number of line crossings and rearing was recorded after 5 min (Smart 3.0, RWD Life Technology, Shenzhen, China), see [41–43] for details. The apparatus was cleaned with 75% alcohol and dried between trials. The experiment was repeated 3 times for each animal.

#### 2.3.3. Pole Test

The pole test can measure the degree of bradykinesia and ability to balance the movement of animals. Mice were placed head up near the top of a wooden pole (2.5 cm in diameter and 55 cm in height). The latency until mice turned completely downward was recorded (defined as turn time, T-turn), and the time taken to reach the floor (locomotor activity time, T-LA) was recorded. Every mouse was tested 3 times.

### 2.4. Western Blot

Mice were sacrificed after anesthetization with 20% urethane. The brains were removed rapidly, and the substantia nigra (SN) and striatum areas were cut into 1 mm coronal cross sections using a vibratome (Leica Microsystems, Wetzlar, Germany), using a brain atlas [44]. The tissue was cryopreserved at −80°C for storage. High efficiency RIPA cracking fluid (R0010, Solarbio, Beijing, China) was added to protease inhibitors and phosphatase inhibitors (P1269, Solarbio, Beijing, China) for tissue lysis. Then, 150 *μ*l lysate was added to every 10 mg tissue. After 30 min, tissue samples were centrifuged at 14,000 rpm for 10 min at 4°C. The protein concentration was quantified by the BCA protein assay (PC0020, Solarbio, Beijing, China). Loading buffer was added to tissue lysates and boiled for 10 min. Equivalent amounts of protein were separated on 10% SDS-polyacrylamide gel and transferred to polyvinylidene difluoride membranes that were blocked with 5% nonfat milk in TBST and then incubated overnight at 4°C with the primary antibody *β*-actin (1 : 2000), Mfn2 (1 : 1000), OPA1 (1 : 1000), Drp1 (1 : 1000), Nrf2 (1 : 1000), (pSer129, SPC-742S, Canada), TNF-*α* (Abcam, ab1793), and HO-1 (1 : 1000). This was followed by incubation for 2 h at room temperature with goat anti-rabbit IgG HRP (1 : 5000) and goat anti-mouse IgG HRP (1 : 5000). The bands were visualized by ECL-enhanced chemiluminescence (Beyotime Institute of Biotechnology, Shanghai, China) according to the manufacturer's instructions. Western blot gels were analyzed with a chemiluminescence imaging system (Thermo Fisher Scientific Inc., Massachusetts, USA) and quantified using ImageJ v1.51 (National Institutes of Health, Bethesda, MD, USA).

### 2.5. Transmission Electron Microscopy (TEM)

Mice were randomly taken from each group. Brain tissues were taken after rapid decapitation after isoflurane anesthesia, fixed in 2.5% glutaraldehyde solution and rinsed, and then placed in 1% osmic acid for fixation. This was followed by alcohol and acetone gradient dehydration, embedding, and immersion polymerization. Ultrathin sectioning was done on a microtome (Leica EM UC7) into 70 nm thick sections and stained with uranyl acetate-lead citrate. The ultrastructure of sections was imaged on a TEM (JEM-1400; JEOL Ltd., Tokyo, Japan) at 60 KV. Five neuropils under 30,000-fold visual fields were photographed, and the average number of synapses was estimated. The thickness of synaptic cleft and postsynaptic membranes density (PSD95 thickness) was evaluated. Data were analyzed by ImageJ v1.51 software (National Institutes of Health, Bethesda, Maryland).

### 2.6. Statistical Analysis

GraphPad Prism 9 software was used for the statistical analysis. The data were analyzed by one-way ANOVA and Tukey post hoc tests. Data are shown as M ± SEM. *P* < 0.05 was considered as significant.

## 3. Results

### 3.1. Rotarod Motor Assessment

**Figure 2:**
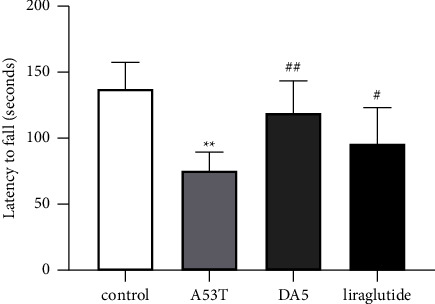
Testing spontaneous motor activity in the rotarod test. DA5-CH reduced the motor impairment better than liraglutide. ^*∗∗*^*P* < 0.01 compared to the control group; ^#^*P* < 0.05 and ^##^*P* < 0.01 compared to the A53T saline-treated group. *N* = 12 per group.

In the rotarod motor coordination assessment, an overall significance in a one-way ANOVA was found (*P* < 0.001). In post hoc tests, the A53T sal group was different from the sal control group (*P* < 0.01), and both liraglutide group and DA5-CH group were better than the A53T sal group (*P* < 0.05), *N* = 12 per group, see Figure 2.

### 3.2. Pole Test

**Figure 3:**
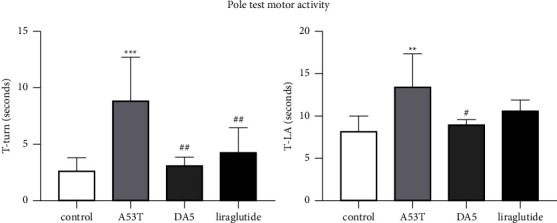
Testing motor activity and response time in the pole test. DA5-CH reduced the motor impairment better than liraglutide. ^*∗∗∗*^*P* < 0.001 and ^*∗∗*^*P* < 0.01 compared to the control group; ^#^ = *P* < 0.05 and ^##^ = *P* < 0.01 compared to the A53T saline-treated group. Control = wild-type saline-treated animals, A53T = A53T saline-treated mice, DA5 = A53T DA5-treated mice, and liraglutide = A53T lira-treated mice. *N* = 12 per group.

In the T-turn assessment, an overall significance in a one-way ANOVA was found (*P* < 0.001). In post hoc tests, the A53T sal group was different from the sal control group (*P* < 0.001), and both liraglutide group and DA5-CH group were better than the A53T sal group (*P* < 0.01) and the DA5-CH group was better than the liraglutide group (*P* < <0.05). In the T-LA evaluation, an overall significance in a one-way ANOVA was found. In post hoc tests, the A53T sal group was different from the sal control group (*P* < 0.01), and the DA5-CH group was better than the A53T sal group (*P* < 0.05), *N* = 12 per group, see Figure 3.

### 3.3. Open Field Test

**Figure 4:**
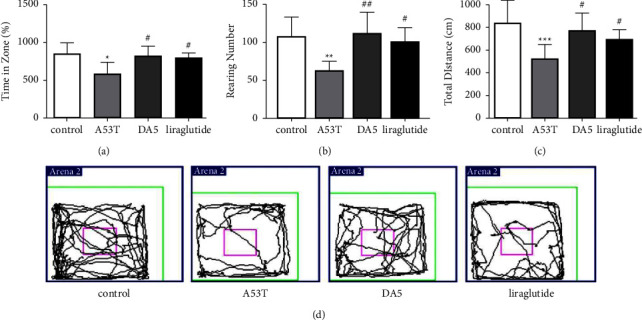
Testing spontaneous motor activity in the open field test. DA5-CH improved exploratory, anxiety, and motor activities better than liraglutide. (a–c) The percentage of residence time in the central area, the total numbers of rearing, and the total distance moved for each group. (d) Track examples of the open field test for each group of mice. ^*∗*^*P* < 0.05, ^*∗∗*^*P* < 0.01, and ^*∗∗∗*^*P* < 0.001 compared to the control group; ^#^*P* < 0.05 and ^##^*P* < 0.01 compared to the A53T saline-treated group. *N* = 12 per group.

In the open field assessment, an overall significance in a one-way ANOVA was found (*P* < 0.001). In the number of explorative rearing, the A53T sal group was different from the sal control group (*P* < 0.01), and the liraglutide group (*P* < 0.05) and DA5-CH group (*P* < 0.01) were better than the A53T sal group (*P* < <0.05). In the time in central zone anxiety evaluation, an overall significance in a one-way ANOVA was found. In post hoc tests, the A53T sal group was different from the sal control group (*P* < 0.01), and the DA5-CH group was better than the A53T sal group (*P* < 0.01), while the liraglutide group was better only at *P* < 0.05. In the total distance evaluation, an overall significance in a one-way ANOVA was found. In post hoc tests, the A53T sal group was different from the sal control group (*P* < 0.01), and the DA5-CH group and the liraglutide group were better than the A53T sal group (*P* < 0.05), *N* = 12 per group, see Figure 4.

### 3.4. Levels of *α*-Synuclein in the Substantia Nigra

**Figure 5:**
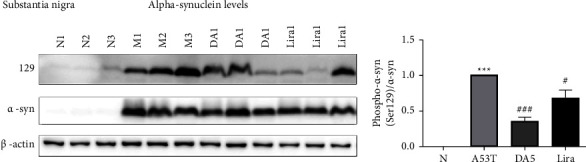
Levels of *α*-synuclein in the substantia nigra. DA5 was better than liraglutide in lowering levels. ^*∗∗∗*^*P* < 0.001 compared to the control group; ^#^*P* < 0.05 and ^###^*P* < 0.001 compared to the A53T saline-treated group. *N* = 5-6 per group.

When analyzing the levels of *α*-syn found in the substantia nigra, an overall significance in a one-way ANOVA was found (*P* < 0.001). The A53T sal group had higher levels than the sal control group (*P* < 0.001), and the liraglutide group (*P* < 0.05) and DA5-CH group (*P* < 0.001) were better than the A53T sal group. DA5-CH was more effective in lowering *α*-syn levels than liraglutide (*P* < 0.05), see Figure 5.

### 3.5. Levels of Proinflammatory Cytokines in the Substantia Nigra

**Figure 6:**
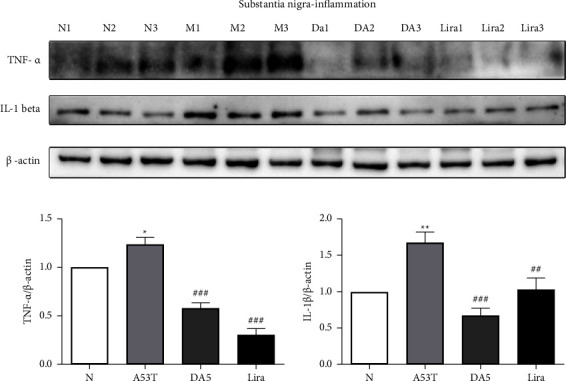
Levels of proinflammatory cytokines in the substantia nigra. DA5 lowered IL-1*ß* levels more than liraglutide. ^*∗∗*^*P* < 0.01 compared to the control group; ^##^*P* < 0.01 and ^###^*P* < 0.001 compared to the A53T saline-treated group. *N* = 5-6 per group.

When analyzing the levels of proinflammatory cytokines in the substantia nigra, an overall significance in a one-way ANOVA was found (*P* < 0.001). TNF-*α*: the A53T sal group had higher levels than the sal control group (*P* < 0.05). The liraglutide group and the DA5-CH group were both lower than the A53T sal group (*P* < 0.001). IL-1*ß*: the A53T sal group had higher levels than the sal control group (*P* < 0.01). The liraglutide group (*P* < 0.01) and the DA5-CH group (*P* < 0.001) were both lower than the A53T sal group. DA5-CH was more effective in lowering IL-1*ß* levels than liraglutide, see Figure 6.

### 3.6. Levels of Pro- and Anti-Inflammatory Cytokines in the Striatum

**Figure 7:**
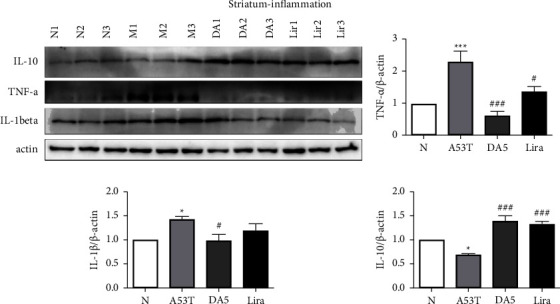
Levels of proinflammatory cytokines in the striatum. DA5 lowered TNF-*α* and IL1*ß* levels more than liraglutide. DA5-CH increased levels of anti-inflammatory IL-10 than liraglutide. ^*∗*^*P* < 0.05 and ^*∗∗∗*^*P* < 0.001 compared to the control group; ^#^*P* < 0.05 and ^###^*P* < 0.001 compared to the A53T saline-treated group. *N* = 5-6 per group.

When analyzing the levels of proinflammatory cytokines in the striatum, an overall significance in a one-way ANOVA was found (*P* < 0.001). TNF-*α*: the A53T sal group had higher levels than the sal control group (*P* < 0.001). The liraglutide group (*P* < 0.05) was not as effective as the DA5-CH group (*P* < 0.001) in lowering levels compared to the A53T sal group. IL-1*ß*: the A53T sal group had higher levels than the sal control group (*P* < 0.05). The DA5-CH group (*P* < 0.05) was lower than the A53T sal group, while the liraglutide group showed no difference. The DA5-CH was more effective in lowering cytokine levels than liraglutide. IL-10 levels: the A53T sal group had lower levels than the sal control group (*P* < 0.05). The liraglutide group and the DA5-CH group were both higher than the A53T sal group (*P* < 0.001), see Figure 7.

### 3.7. Levels of Autophagy Biomarkers in the Substantia Nigra

**Figure 8:**
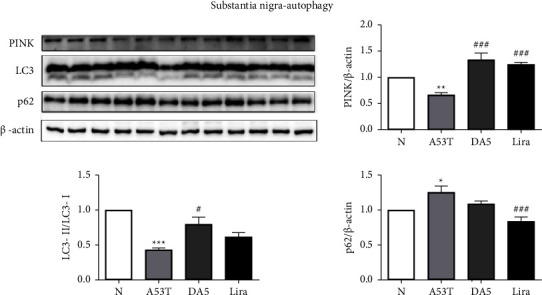
Levels of autophagy-related proteins in the substantia nigra. DA5 normalized levels more than liraglutide. ^*∗∗*^*P* < 0.01 compared to the control group; ^*∗∗∗*^*P* < 0.001 compared to the control group; ^#^*P* < 0.05, ^##^*P* < 0.01, and ^###^*P* < 0.001 compared to the A53T saline-treated group. *N* = 5-6 per group.

When analyzing the levels of autophagy-related proteins in the substantia nigra, an overall significance in a one-way ANOVA was found (*P* < 0.001). PINK1 levels: the A53T sal group had lower levels than the sal control group (*P* < 0.01). The liraglutide group and the DA5-CH group were both lower than the A53T sal group (*P* < 0.001). LC3-II/LC3-1: the A53T sal group had higher levels than the sal control group (*P* < 0.01). The DA5-CH group (*P* < 0.05) had lower levels than the A53T sal group. DA5-CH was more effective in lowering levels than liraglutide. P62: the A53T sal group had higher levels than the sal control group (*P* < 0.05). The liraglutide group (*P* < 0.05) had lower levels than the A53T sal group, see Figure 8.

### 3.8. TEM Analysis of Mitochondria and Autophagosome Morphology

**Figure 9:**
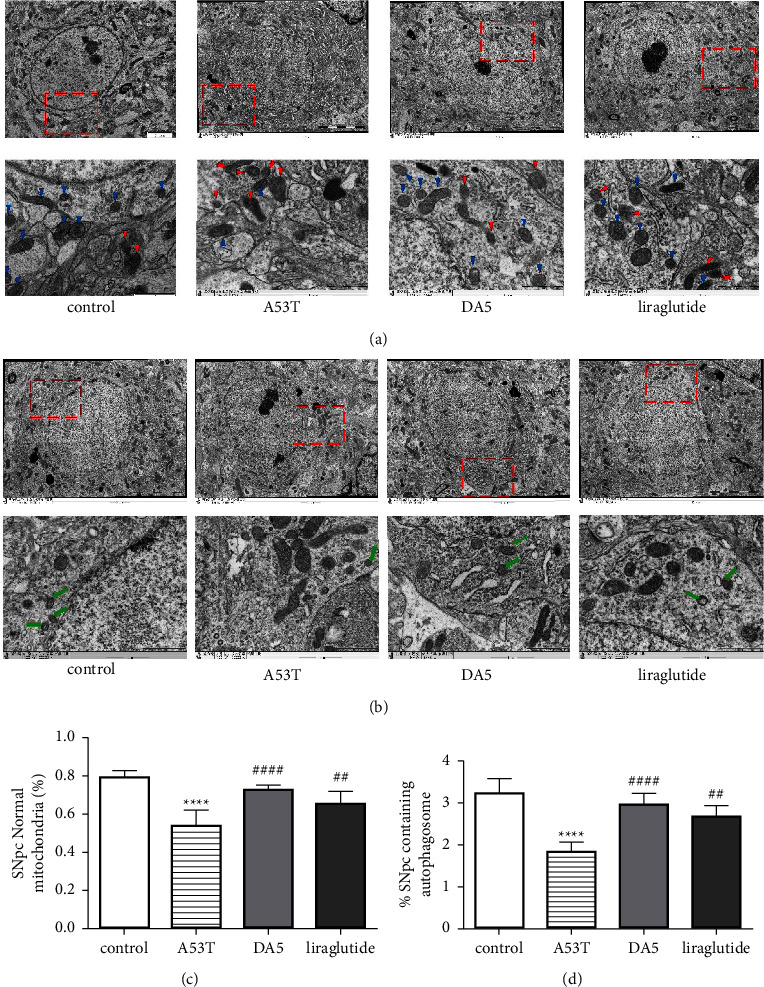
(a) TEM observation of mitochondria in the SNpc of mice in each group. The blue triangles represent normal mitochondria and the red triangles represent damaged mitochondria (scale bar: 2 *μ*m and 1 *μ*m). (b) TEM observation of autophagosomes (green arrows) in the SNpc of mice in each group (scale bar: 2 *μ*m and 1 *μ*m). (c) Quantification of (a). (d) Quantification of (b). ^*∗∗∗∗*^*P* < 0.0001 compared to the control group; ^##^*P* < 0.01 and ^####^*P* < 0.0001 compared to the A53T group. *N* = 5-6 per group.

The shapes of mitochondria and autophagosomes were analyzed in TEM images. Changes in morphology that suggest damage have been observed in the A53T mouse brain tissue compared to wild-type controls (*P* < 0.0001). Drug treatment normalized these changes, and DA5-CH treatment showed a difference compared to the A53T tissue (*P* < 0.001), while liraglutide showed a difference of *P* < 0.01 compared to the A53T tissue. There was no difference between drug group values and wild-type controls, see Figure 9.

## 4. Discussion

Type 2 diabetes mellitus (T2DM) is a risk factor for developing PD [45–49]. Insulin desensitization was found in the brains of people who had PD, even if they did not have diabetes [7, 50]. There is an additional association between insulin resistance and an increased risk of PD dementia, a more severe PD phenotype [51]. Insulin desensitization is found in the brains of PD animal models independently of diabetes, too [52, 53]. Therefore, drugs originally developed to treat T2DM that reduce insulin resistance have been tested in animal models of PD and in clinical trials [8, 13, 26]. Importantly, a phase 2 clinical trial that tested the GLP-1 receptor agonist exendin-4 (Bydureon) showed improvements in PD patients (NCT01971242). After 48 weeks of drug treatment, the motor activity was improved compared to placebo treatment, and the improvement remained visible 12 weeks later. DAT brain imaging showed a reduced deterioration of the dopaminergic nigral-striatal system [14, 54]. When analyzing neuronal exosomes in these patients, it was found that insulin sensitivity has been improved in the brain by the drug [16]. A second phase 2 trial testing the GLP-1 analogue liraglutide (Victoza) showed meaningful improvements in everyday motor activities such as walking, chewing, talking, and getting out of a chair compared to placebo treatment. An improvement in the quality of life score was observed, too [21].

Liraglutide and other GLP-1 receptor agonists have been developed to treat T2DM and remain in the blood stream for a long time [12, 55]. Therefore, the ability to cross the blood-brain barrier (BBB) to get into the brain is limited [27, 56, 57]. In order to improve target engagement, drugs need to be able to get into the brain. There is a direct correlation between crossing the BBB and protecting the CNS [27, 28, 32, 58]. We, therefore, developed novel drugs that can cross the BBB at an enhanced rate [25, 32, 59]. Exendin-4 can cross the BBB well and showed good effects in animal models of PD [27, 60] and in the clinic [14, 61]. In contrast, the company Neuraly developed a PEGylated version of exendin-4 as a novel treatment for PD [62], but this version with a 40 kDa PEGylation attached does not readily cross the BBB and was inferior in a direct comparison with DA5-CH in the MPTP mouse model of PD [32]. Importantly, it did not show any improvements in a phase II clinical trial in PD patients either [31]. DA5-CH in contrast has been developed to cross the BBB at an enhanced rate [27, 40, 63].

Glucose-dependent insulinotropic polypeptide (GIP) is the sister incretin hormone of GLP-1 [64]. GIP analogues have similar protective properties as GLP-1 has in animal models of PD [24, 65, 66]. Our dual GLP-1/GIP receptor agonist DA5-CH (KP405) has better neuroprotective effects in the MPTP mouse model of PD when directly compared to liraglutide [25, 28] and is furthermore more effective than exendin-4 in the 6-OHDA rat model of PD [63]. Both exendin-4 (Bydureon) and liraglutide (Victoza) have shown good protective effects in clinical trials in PD [14, 16, 21]. In the present study, we show that DA5-CH is superior to liraglutide in the A53T mouse model of PD, too.

GLP-1 and GIP both act as growth factors in the brain and improve a range of key pathological developments in the PD brain. Insulin signalling is reduced, glucose utilization and energy production are impaired, mitochondrial activities such as mitophagy and mitogenesis are compromised, autophagy and the removal of aggregated proteins are normalized, gene expression of key growth factors such as BDNF and GDNF is normalized, dopamine synthesis and synaptic activity are improved, and the chronic inflammation response is reduced, see [13, 59, 65, 67] for details. The mechanism of action is a single process rather than the sum of all of these improvements. Importantly, GLP-1 and GIP act synergistically and therefore show enhanced protective effects in comparison with a single GLP-1 receptor agonist [68, 69]. Chronic inflammation in the brain is observed in PD, and it plays a key role in disease progression [70, 71]. Initial triggers such as toxins can active microglial cells which release proinflammatory cytokines and free radicals. This starts a chronic neuroinflammatory process that can kill vulnerable neuronal populations such as dopaminergic neurons [5, 72]. Importantly, proinflammatory cytokines can induce insulin desensitization and reduce growth factor synthesis and function [73–76]. Our study demonstrates that a chronic inflammation response is present in the brains of A53T mice, and liraglutide and DA5-CH downregulate TNF-*ɑ* and IL-1*ß* levels in the substantia nigra. This result confirms our previous study findings in the MPTP mouse model of PD, where chronic inflammation in the brain was much reduced by liraglutide [19] and DA5-CH [28, 32, 63]. The reduction of chronic inflammation by these drugs is driven by activating the GLP-1 receptor on microglia, which reduces the inflammation as GLP-1 also acts as an anti-inflammatory cytokine [77, 78]. The reduction of TNF-*ɑ* and IL-1*ß* levels in the brain will contribute to the reversal of insulin desensitization in the brain. TNF-*α* can reduce IRS-1 serine phosphorylation, which blocks the second messenger cascade activated by the insulin receptor [73, 79]. We showed in previous studies that liraglutide or DA5-CH reduced the tyrosine 312 phosphorylation of IRS-1 in the 6-OHDA rat model of PD [29]. This demonstrates that the drugs can reactivate insulin signalling which was blocked by the chronic inflammation response. Other studies have shown similar insulin resensitization with GLP-1 class drugs [11, 30, 80, 81].

**Figure 10:**
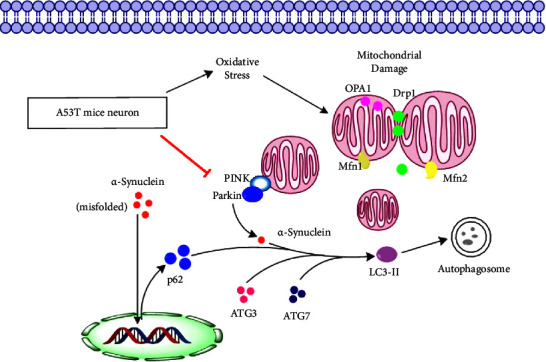
Overview of the proposed mechanism of clearance of *α*-synuclein. Autophagy is compromised in the A53T tg mouse model. Activating the GLP-1 and GIP receptors increases autophagy in neurons via CREB signalling, thereby reducing the levels of *α*-synuclein in the substantia nigra and the striatum.

The A53T mouse model expresses a human-mutated gene and is a standard animal model for *α*-syn proteinopathy [39, 82]. A leading hypothesis is that “misfolding” and aggregation of *α*-synuclein, a component of Lewy bodies in frontotemporal dementia (FTD) patients, are driving PD progression [83]. The reason why *α*-syn accumulates and aggregates in the brain is believed to be overexpression of the protein and failure to remove the protein by autophagy [84, 85]. Furthermore, misfolding of *α*-syn and formation of oligomers and fibrils have shown to interfere with normal cellular processes which ultimately lead to neuronal death [33–35]. The cerebrospinal fluid (CSF) of PD patients can contain increased levels of *α*-syn oligomers [86]. However, other studies could not detect these oligomers in brain sections. It was found that while *α*-syn oligomers can be neurotoxic, Lewy bodies, the fibrillar form of *α*-syn, can be neuroprotective [87]. Clinical trials testing antibodies that reduce the levels of *α*-syn in the brain did not show improvements in PD patients [88–90]. A potential mechanism of how *α*-syn oligomer could induce neurodegeneration in PD may include the disruption of a variety of cellular processes, such as mitochondrial impairments, endoplasmic reticulum stress, synaptic functions, dysfunction of the autophagy pathway, and activating microglia [91]. Importantly, both liraglutide and DA5-CH reduced the levels of *α*-syn in the substantia nigra. The dual agonist DA5-CH was more effective. This result confirms a previous result measuring *α*-syn monomers and oligomers found in the 6-OHDA rat model of PD [29]. The underlying mechanism for reducing the levels of *α*-syn is most likely the normalization of autophagy, which can remove the protein. We and others have shown that GLP-1 class drugs can normalize autophagy and remove proteins such as *α*-syn or *ß*-amyloid that can accumulate and aggregate in the brain [92–96], for a detailed review, see [67]. We were able to show in our study that drug treatment can normalize levels of proteins that play key roles in autophagy, which supports this concept, see Figure 10. Again, the dual agonist was more effective than liraglutide.

Mitochondrial dysfunction is a key pathological feature of PD [97–99]. GLP-1 and GIP mimetics can improve mitochondrial activities in animal models of PD [20, 24, 92, 100], and DA5-CH improved mitochondrial activities, too [13, 67]. The present study shows that the expression levels of key mitochondrial proteins and the morphology of mitochondria are normalized, too.

As liraglutide has already shown meaningful improvements in a phase 2 clinical trial of PD patients [21], DA5-CH may be more effective than liraglutide. In addition, we recently finished a phase 2 clinical trial testing liraglutide in patients with Alzheimer's disease. Here, the drug improved the scores in tests of cognition and slowed down brain shrinkage as shown in MRI scans [101, 102]. Therefore, DA5-CH may be an effective treatment for this disease, too. A phase 1 clinical trial of DA5-CH (KP405) has started.

## 5. Conclusion

This study demonstrates good effects of the dual GLP-1/GIP receptor agonist DA5-CH (KP405) in the A53T mouse model of PD. Motor impairments were alleviated, and levels of *α*-synuclein and proinflammatory cytokines were much reduced in the brain, and proteins that play key roles in the process of autophagy and mitochondrial activity were brought back to physiological levels. In comparison, liraglutide was not as effective in alleviating these pathological features.

## Data Availability

The data used to support the findings of this study are included within the manuscript. Further information is available from the corresponding authors on request.
